# Profiling Differential Effects of 5 Selective Serotonin Reuptake Inhibitors on TLRs-Dependent and -Independent IL-6 Production in Immune Cells Identifies Fluoxetine as Preferred Anti-Inflammatory Drug Candidate

**DOI:** 10.3389/fphar.2022.874375

**Published:** 2022-06-22

**Authors:** Yohei Takenaka, Ryu Tanaka, Kazuki Kitabatake, Kouji Kuramochi, Shin Aoki, Mitsutoshi Tsukimoto

**Affiliations:** ^1^ Department of Radiation Biosciences, Faculty of Pharmaceutical Sciences, Tokyo University of Science, Chiba, Japan; ^2^ Department of Applied Biological Science, Faculty of Science and Technology, Tokyo University of Science, Chiba, Japan; ^3^ Department of Bioorganic and Bioinorganic Chemistry, Faculty of Pharmaceutical Sciences, Tokyo University of Science, Chiba, Japan; ^4^ Research Institute for Science and Technology (RIST), Tokyo University of Science, Chiba, Japan

**Keywords:** selective serotonin reuptake inhibitor, IL-6, cytokine, macrophage, dendritic cells, lymphocytes, Toll-like receptor, concanavalin A

## Abstract

Excessive proinflammatory cytokine production induced by abnormal activation of Toll-like receptor (TLR) signaling, for example, by SARS-CoV-2 infection, can cause a fatal cytokine storm. The selective serotonin reuptake inhibitors (SSRIs) fluoxetine and fluvoxamine, used to treat depression, were recently reported to reduce the risk of severe disease in patients with coronavirus disease 2019 (COVID-19), but the mechanisms of the anti-inflammatory effects of SSRIs, and which SSRI would be most suitable as an anti-inflammatory drug, remain unclear. Here, we examined the inhibitory effects of 5 FDA-approved SSRIs, paroxetine, fluoxetine, fluvoxamine, sertraline and escitalopram, on the production of interleukin-6 (IL-6) induced by stimulation with multiple TLR agonists in murine macrophages and dendritic cells, and on the production of cytokines induced by concanavalin A in murine lymphocytes. In J774.1 murine macrophage cells, pretreatment with SSRIs significantly suppressed IL-6 release induced by TLR3 agonist poly(I:C), TLR4 agonist LPS or TLR9 agonist CpG ODN, but did not affect IL-6 release induced by TLR7 agonists imiquimod or resiquimod. In accordance with the results obtained in J774.1 cells, pretreatment with SSRIs also suppressed IL-6 release induced by a TLR3, TLR4 or TLR9 agonist in bone marrow-derived dendritic cells and peritoneal cells of C57BL/6 mice. On the other hand, interestingly, sertraline alone among the SSRIs amplified IL-6 production induced by TLR7 agonists in murine dendritic cells, though not in macrophages. Concanavalin A-induced production of IL-6 or IL-2 in murine lymphocytes was suppressed by SSRIs, suggesting that SSRIs also inhibit TLRs-independent IL-6 production. Since SSRIs suppressed both IL-6 production induced by multiple TLR agonists in macrophages or dendritic cells and TLR-independent IL-6 production in lymphocytes, they are promising candidates for treatment of patients with cytokine storm, which is mediated by overactivation of multiple TLRs in a complex manner, leading to the so-called IL-6 amplifier, an IL-6 overproduction loop. However, the 5 SSRIs examined here all showed different effects. Overall, our results suggest that fluoxetine may be the most promising candidate as an anti-inflammatory drug. An examination of the structural requirements indicated that the *N*-methyl group of fluoxetine has a critical role in the inhibition of IL-6 production.

## Introduction

Sepsis is a systemic inflammatory syndrome triggered by infections caused by bacteria, viruses, fungi and parasites. It is a potentially fatal disease caused by excessive proinflammatory cytokine production (cytokine storm) induced by abnormal activation of Toll-like receptor (TLR) signaling (Opal et al., 2013; Chousterman et al., 2017; Kuzmich et al., 2017; Crayne et al., 2019; Shimizu, 2020). In 2017, 11.1 million sepsis-related deaths were recorded, representing 19.7% of all deaths worldwide (Rudd et al., 2020). The pathogenesis of sepsis is diverse, and effective therapeutic strategies are still needed. We hypothesized that drugs which inhibit the cytokine storm involved in the exacerbation of sepsis would be effective therapeutic agents. The current major therapeutic agents for cytokine storms are steroidal anti-inflammatory drug (dexamethasone) and anti-human interleukin-6 (IL-6) receptor monoclonal antibody (tocilizumab) (Shimizu, 2020; Gordon et al., 2021). However, the former has serious side effects such as increased risk of infection, and the latter is an expensive biopharmaceutical. A TLR4 antagonist (Eritoran (E5564)), which has been proposed as a potential therapeutic agent for sepsis, failed to show a significant therapeutic effect, probably because multiple TLRs signals are activated in patients (Opal et al., 2013). We noted that IL-6 amplifier, an IL-6 overproduction loop involving activation of nuclear factor-kappa B (NF-κB) and signal transducer and activator of transcription 3 (STAT3), is regarded as the main culprit in cytokine storms (Matsuyama et al., 2020), so we considered it plausible that drugs inhibiting the production of proinflammatory cytokines (especially IL-6) induced by the activation of multiple TLRs signals would be effective therapeutic agents.

Innate immunity is the first defense against pathogens such as bacteria, viruses and parasites, serving as a risk-sensing system. This system utilizes pattern recognition receptors (PRRs), such as TLRs, to recognize pathogen-associated molecular patterns (PAMPs) released from foreign microorganisms. For example, TLR4 recognizes lipopolysaccharide (LPS) released from gram-negative bacteria, while TLR3, 7, 8 and 9 recognize viral nucleic acids (Chen et al., 2017; Chousterman et al., 2017; Kuzmich et al., 2017; Briard et al., 2020). Recent reports show that TLRs recognize not only PAMPs, but also damage-associated molecular patterns (DAMPs), and TLRs are involved in not just infections, but also the exacerbation of chronic inflammation, which is a feature of depression, cancer, rheumatoid arthritis, and Alzheimer’s disease (Hung et al., 2016; Kuzmich et al., 2017). Thus, drugs with the ability to inhibit TLR signaling are considered to be candidate therapeutic agents not only for infection-related diseases, but also for various inflammatory diseases.

The US Food and Drug Administration (FDA) has so far approved a number of selective serotonin reuptake inhibitors (SSRIs), i.e., paroxetine, fluoxetine, fluvoxamine, sertraline, citalopram, escitalopram (*S*-form of the optical isomers of citalopram), vilazodone (also a partial agonist of 5-HT_1A_ receptor), and vortioxetine (also an agonist of 5-HT_1A_ receptor and an antagonist of 5-HT_3_ receptor) for the treatment of depression (Hiemke and Härtter, 2000; Lochmann and Richardson, 2019). Recently, it has been reported that SSRIs decrease the levels of inflammatory factors in the blood of patients taking them (Hung et al., 2016; Więdłocha et al., 2018), and that fluoxetine and fluvoxamine reduce the risk of severe disease in patients with coronavirus disease 2019 (COVID-19) (Lenze et al., 2020; Németh et al., 2021; Reis et al., 2022). However, details of the mechanism of the anti-inflammatory effects of SSRIs remain to be established. Also, it is not clear which SSRI would be the most suitable candidate for an anti-inflammatory drug, especially for treating cytokine storms.

In this study, we profiled the effects of 5 FDA-approved SSRIs, paroxetine, fluoxetine, fluvoxamine, sertraline and escitalopram, on IL-6 production induced by various TLR agonists, i.e., poly(I:C) (TLR3 agonist), LPS (TLR4 agonist), imiquimod and resiquimod (TLR7 agonists), and CpG ODN (TLR9 agonist), in various immune cells (J774.1 murine macrophage cells, bone marrow-derived dendritic cells (BMDCs) and murine peritoneal cells) and on IL-6/IL-2 production induced by concanavalin A (T cell activator) independently of TLRs in murine splenic lymphocytes. Our results show that these SSRIs all significantly suppress IL-6 production induced by multiple TLR agonists or T cell activator, but there were significant differences in the effects of the 5 drugs on IL-6 production induced by stimulation with agonists of TLR3, TLR4, TLR7 and TLR9. Our findings support the idea that SSRIs might be useful as anti-inflammatory drugs to suppress excessive production of cytokines such as IL-6, and also suggest that fluoxetine may be the most promising candidate for further evaluation.

## Materials and Methods

### Reagents

Paroxetine, fluoxetine, fluvoxamine and sertraline (SSRIs) were purchased from FUJIFILM Wako Pure Chemical Corporation (Osaka, Japan). Escitalopram (an SSRI) was purchased from Tokyo Chemical Industry Co., Ltd. (Tokyo, Japan). Polyinosinic-polycytidylic acid (poly(I:C)) (a TLR3 agonist) and 5-(3-bromophenyl)-1,3-dihydro-2H-benzofuro[3,2-e]-1,4-diazepin-2-one (5-BDBD) (a selective antagonist of P2X4 receptor) were purchased from Tocris Bioscience (Minneapolis, MN, United States). Lipopolysaccharide (LPS) from *Escherichia coli* O55:B5 (a TLR4 agonist) and concanavalin A (ConA) (a T cell activator) were purchased from Sigma-Aldrich Co. LLC (St. Louis, MO, United States). Imiquimod (R837) (a TLR7 agonist) was purchased from Chemscene (Monmouth Junction, United States). Resiquimod (R848) (a TLR7/8 agonist) was purchased from AdipoGen Life Sciences (Liestal, Switzerland). CpG oligodeoxynucleotides (ODN; 1826) (a TLR9 agonist) was purchased from Novus Biologicals (Centennial, United States). 10-[(4-Methylphenyl)sulfonyl]-10*H*-phenoxazine (PSB12062) (a selective antagonist of P2X4 receptor) was purchased from Glixx Laboratories (Southborough, United States). 1-[2-(3,4-Dichlorophenyl)ethyl]-4-methylpiperazine (BD1063) (a selective antagonist of sigma-1 receptor) was purchased from abcam plc (Cambridge, United Kingdom). 5-Hydroxytryptamine (serotonin) was purchased from Cayman Chemical (MI, United States).

### Cell Culture

J774.1 cells (RIKEN BioResource Center, Ibaraki, Japan) were grown in RPMI 1640 medium (FUJIFILM Wako Pure Chemical Corporation, Osaka, Japan), supplemented with 10% heat-inactivated fetal bovine serum (FBS) (Gibco, MA, United States), 100 U/ml penicillin and 100 μg/ml streptomycin in a humidified atmosphere of 5% CO_2_ in air at 37°C.

### Animals

The mice were housed as described previously (Sakaki et al., 2013; Shinohara and Tsukimoto, 2017). Male C57BL/6 mice were purchased from Sankyo Labo Service Corporation, Inc. (Tokyo, Japan) and used at 6 weeks of age. The mice were treated and handled according to the Tokyo University of Science’s institutional ethical guidelines for animal experiments and with the approval of Tokyo University of Science’s Institutional Animal Care and Use Committee (permission number; S20001, S21006).

### Generation of Dendritic Cells From Bone Marrow

Bone marrow from 6-week-old male C57BL/6 mice was used. The femur and fibula were collected and washed with RPMI-1640 medium to obtain cell suspensions. Differentiation of isolated bone marrow cells into dendritic cells was performed as described previously (Sakaki et al., 2013). Granulocyte-macrophage colony stimulating factor (GM-CSF), a differentiation inducer, was purchased from BioLegend (San Diego, CA, United States).

### Preparation of Peritoneal Cells

Male C57BL/6 mice (6 weeks old) were euthanized, and the peritoneal cavity was washed with ice-cold sterile PBS and the cell-containing fluid was collected. The collected cells were washed twice with RPMI-1640 medium, seeded in 96-well plates, and then incubated with the test reagents for 24 h in an atmosphere of 5% CO_2_/95% air at 37°C.

### Preparation of Splenic Lymphocytes

The lymphocytes were prepared from mouse spleen as described previously (Shinohara and Tsukimoto, 2017).

### Enzyme-Linked Immunosorbent Assay

J774.1 cells or peritoneal cells isolated from 6-week-old male C57BL/6 mice were pre-incubated with each SSRI (1–10 µM) for 30 min, then incubated with poly(I:C) (5 μg/ml), LPS (1 μg/ml), CpG ODN (200 ng/ml), imiquimod (1 μg/ml) or resiquimod (10 ng/ml) for 6 or 24 h in an atmosphere of 5% CO_2_ in air at 37°C. Murine BMDCs were pre-incubated with each SSRI (1–10 µM) for 30 min, then incubated with poly(I:C) (10 μg/ml), LPS (1 μg/ml), CpG ODN (200 ng/ml), imiquimod (1 μg/ml) or resiquimod (10 ng/ml) for 24 h in an atmosphere of 5% CO_2_ in air at 37°C. Murine splenic lymphocytes were pre-incubated with each SSRI (1–10 µM) for 30 min, then incubated with ConA (5 μg/ml) for 24 h in an atmosphere of 5% CO_2_ in air at 37°C.

Culture supernatant was harvested, and IL-6 or IL-2 was measured by ELISA. 96-well plates were coated with purified anti-mouse IL-6 monoclonal antibody (mAb) (1:1,000) or purified anti-mouse IL-2 mAb (1:1,000) (eBioscience, San Diego, CA, United States). The plates were incubated at 4°C overnight, washed with PBS containing 0.05% Tween-20, and blocked with PBS containing 1% bovine serum albumin (BSA). The plates were incubated for 1 h at room temperature, washed again, and then incubated overnight at 4°C with culture supernatant and recombinant mouse IL-6 (BioLegend, San Diego, CA, United States) or IL-2 (TONBO Biosciences, San Diego, CA, United States), in order to obtain a standard curve. The plates were washed again and incubated with anti-mouse biotin-conjugated IL-6 mAb (1:1,000) or anti-mouse biotin-conjugated IL-2 mAb (1:1,000) (eBioscience, San Diego, CA, United States) for 1 h at room temperature, then washed and incubated with avidin-horseradish peroxidase (FUJIFILM Wako Pure Chemical Corporation, Osaka, Japan) for 10 min at room temperature. The plates were washed again, and 3,3′,5,5′-tetramethylbenzidine (TMB) (FUJIFILM Wako Pure Chemical Corporation, Osaka, Japan) was added. When an appropriate color reaction was observed, 2.5 M H_2_SO_4_ was added to stop the reaction. The absorbance at 450 nm was measured with a Wallac 1420 ARVO Fluoroscan (Wallac, Turku, Finland).

### Real-Time RT-PCR

RT^2^-qPCR^®^ primer assays for mouse IL-6 and IFN-β were purchased from Qiagen (Venlo, Netherlands). J774.1 cells were pre-incubated with SSRIs (1–10 µM) for 30 min, then incubated with poly(I:C) (5 μg/ml) for 3 h in an atmosphere of 5% CO_2_ in air at 37°C. Total RNA was extracted from J774.1 cells using a ReliaPrep™ RNA Cell Miniprep System (Promega, Madison, WI, United States). IL-6 and IFN-β mRNA expression levels were measured as described previously (Ninomiya et al., 2017).

### Western Blotting

J774.1 cells were pre-incubated with each SSRI (10 µM) for 30 min, then incubated with poly(I:C) (5 μg/ml) for 45 min in an atmosphere of 5% CO_2_ in air at 37°C. J774.1 cells were lysed in cold lysis buffer containing 1% Triton X-100, protease inhibitor cocktail (Sigma-Aldrich Co. LLC, St. Louis, MO, United States) and PhosSTOP at 4°C for 30 min. The lysate was centrifuged at 10,000 × g for 15 min to separate the nuclei, dissolved in sample buffer solution (FUJIFILM Wako Pure Chemical Corporation, Osaka, Japan) containing 10 mM dithiothreitol, and boiled at 95°C for 10 min to prepare the samples.

Aliquots were subjected to 10% sodium dodecyl sulphate (SDS)-polyacrylamide gel electrophoresis (PAGE) and bands were transferred to polyvinylidene difluoride (PVDF) membranes. The blots were incubated for 1 h at room temperature with TBST (0.1% Tween-20, 10 mM Tris-HCl, 0.1 M NaCl) containing 1% BSA, and then further incubated overnight at 4°C with anti-IκBα (L35A5) mouse mAb (1:1,000) (#4814), p38α MAP kinase (5F11) mouse mAb (1:1,000) (#9217), phospho-p38 MAPK (Thr180/Tyr182) (12F8) rabbit mAb (1:1,000) (#4631) (Cell Signaling Technology, Inc., Danvers, Massachusetts, United States), or anti-β-actin mouse mAb (FUJIFILM Wako Pure Chemical Corporation, Osaka, Japan), which was used as a loading control. The blots were washed with TBST, and then incubated with anti-rabbit IgG, HRP-linked antibody (1:10,000) or anti-mouse IgG, HRP-linked antibody (1:20,000) (Cell Signaling Technology, Inc., Danvers, MA, United States) for 1 h at room temperature and washed with TBST. Finally, we detected specific proteins by using ImmunoStar^®^ LD (FUJIFILM Wako Pure Chemical Corporation, Osaka, Japan). Western blotting detection system was from LI-COR, Inc. (Lincoln, NE, United States), and bands were analyzed with Image Studio 4.0 for C-DiGit Scanner (LI-COR, Inc., Lincoln, NE, United States).

### 3-(4,5-Dimethylthiazol-2-yl)-2,5-diphenyltetrazolium Bromide (MTT) Assay

J774.1 cells or BMDCs were seeded in 96-well plates in RPMI-1640 medium and incubated for 24 h. The cells were incubated with SSRIs (1–10 µM) for 24 h, then incubated with MTT (0.5 mg/ml) for 3 h, and the purple formazan crystals generated by mitochondrial dehydrogenase-mediated reduction in each well were dissolved in stop solution. The absorbance of each well was read on a WALLAC 1420 ARVO MX multilabel counter (570 nm).

### Preparation of Fluoxetine Derivatives (FLX-D-1, 2, and 3)

FLX-D-1, 2, and 3 were synthesized from 3-chloro-1-phenylpropan-1-one according to the protocol reported by Silvestri and coworkers (Silvestri et al., 2004). The synthetic products were fully characterized by infrared spectroscopy (IR), ^1^H and ^13^C nuclear magnetic resonance (NMR), and high-resolution mass spectrometry (HRMS).

FLX-D-1: Colorless oil; IR (neat) ν_max_ = 3687, 3028, 2958, 2929, 2858, 1614, 1518, 1454, 1329, 1252, 1217, 1179, 1163, 1122 cm^−1^; ^1^H NMR (400 MHz, CDCl_3_) δ 7.43 (d, *J* = 8.4 Hz, 2H), 7.32 (m, 4H), 7.27 (m, 1H), 6.90 (d, *J* = 8.4 Hz, 2H), 5.31 (dd, *J* = 8.0, 4.8 Hz, 1H), 2.80 (t, *J* = 7.2 Hz, 2H), 2.63 (t, *J* = 7.2 Hz, 2H), 2.23 (m, 1H), 2.06 (m, 1H), 1.48 (m, 2H), 1.27 (m, 6H), 0.87 (t, *J* = 7.0 Hz, 3H); ^13^C NMR (100 MHz, CDCl_3_) δ 160.4, 140.9, 128.8 (2C), 127.8, 126.7 (q, *J* = 4 Hz, 2C), 125.7 (2C), 124.3 (q, *J* = 269 Hz), 122.7 (q, *J* = 32 Hz), 115.7 (2C), 78.6, 49.9, 46.0, 38.5, 31.7, 27.0, 22.6, 14.0; HRMS (ESI/QTOF) *m*/*z*: [M + H]^+^ Calcd for C_22_H_29_F_3_NO 380.2196; Found 380.2196.

FLX-D-2: Colorless oil; IR (neat) ν_max_ = 3020, 2962, 2933, 2873, 2808, 1616, 1518, 1329, 1254, 1215, 1176, 1163, 1122, 1113 cm^−1^; ^1^H NMR (400 MHz, CDCl_3_) δ 7.41 (d, *J* = 8.4 Hz, 2H), 7.34 (m, 4H), 7.26 (m, 1H), 6.90 (d, *J* = 8.4 Hz, 2H), 5.31 (dd, *J* = 8.8, 4.4 Hz, 1H), 2.64 (m, 1H), 2.51 (m, 1H), 2.35 (m, 4H), 2.09 (m, 1H), 1.93 (m, 1H), 1.40 (m, 4H), 0.84 (t, *J* = 7.2 Hz, 6H); ^13^C NMR (100 MHz, CDCl_3_) δ 160.7, 141.5, 128.7 (2C), 127.6, 126.6 (q, *J* = 4 Hz, 2C), 125.8 (2C), 124.4 (q, *J* = 269 Hz), 122.5 (q, *J* = 32 Hz), 115.7 (2C), 78.3, 56.2 (2C), 50.1, 36.7, 20.3 (2C), 12.0 (2C); HRMS (ESI/QTOF) *m*/*z*: [M + H]^+^ Calcd for C_22_H_29_F_3_NO 380.2196; Found 380.2195.

FLX-D-3: Yellow oil; IR (neat) ν_max_ = 3431, 3018, 2927, 1614, 1605, 1516, 1506, 1329, 1250, 1217, 1178, 1163 cm^−1^; ^1^H NMR (400 MHz, CDCl_3_) δ 7.44 (d, *J* = 8.4 Hz, 2H), 7.34 (m, 5H), 7.29 (m, 1H), 7.16 (m, 2H), 6.91 (d, *J* = 8.8 Hz, 2H), 6.71 (tt, *J* = 7.2, 0.8 Hz, 1H), 6.59 (dd, *J* = 8.8, 1.2 Hz, 2H), 5.33 (dd, *J* = 8.4, 4.4 Hz, 1H), 3.33 (m, 2H), 2.31 (m, 1H), 2.17 (m, 1H); ^13^C NMR (100 MHz, CDCl_3_) δ 160.3, 148.0, 140.6, 129.3 (2C), 128.9 (2C), 128.0, 126.8 (q, *J* = 4 Hz, 2C), 125.7 (2C), 124.3 (q, *J* = 269 Hz), 122.9 (q, *J* = 32 Hz), 117.5, 115.7 (2C), 112.8 (2C), 78.5, 40.5, 38.3; HRMS (ESI/QTOF) *m*/*z*: [M + H]^+^ C_22_H_21_F_3_NO ([M + H]^+^) 372.1570; Found 372.1571.

### Statistics

**FIGURE 1:**
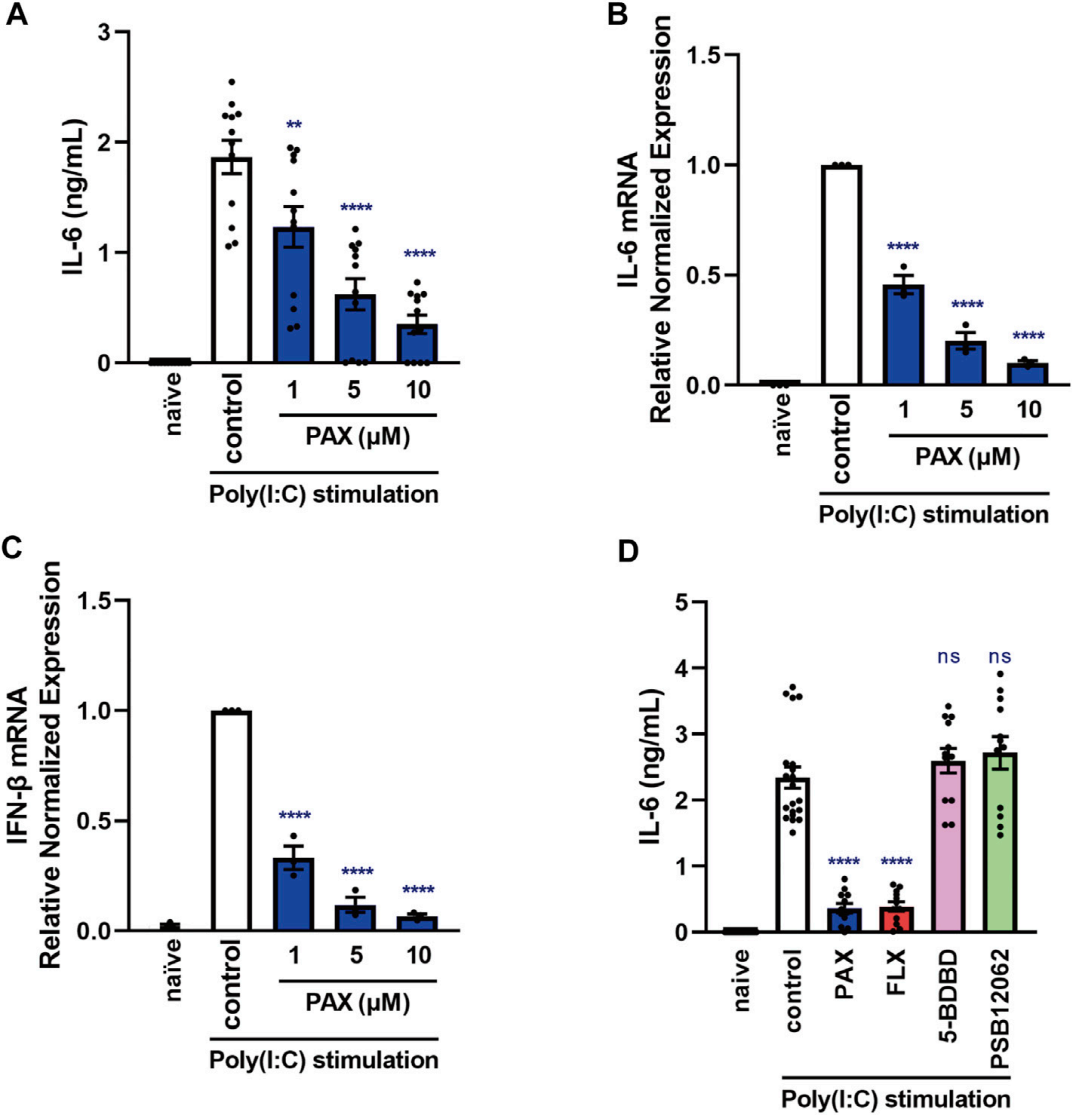
Effects of paroxetine on cytokine production induced by a TLR3 agonist in J774.1 murine macrophage cells. **(A–C)** J774.1 cells were pre-incubated with paroxetine (1–10 µM) for 30 min and then incubated with poly(I:C) (5 μg/ml) for 3 h **(B,C)** or 6 h **(A)**. **(D)** Cells were pre-incubated with paroxetine (10 µM), fluoxetine (10 µM), 5-BDBD (10 µM) or PSB12062 (10 µM) for 30 min and then incubated with poly(I:C) for 6 h. **(A, D)** IL-6 was measured by means of ELISA. **(B,C)** Expression of IL-6 or IFN-β mRNA was analyzed by real-time RT-PCR. Error bars indicate ±SE [**(A)**: *n* = 12, **(B,C)**: *n* = 3, **(D)**: *n* = 12–20, three or more independent experiments]. Statistical analysis was performed by one-way ANOVA followed by Dunnett’s test. Significant differences between control and test groups are indicated by **p < 0.01, ****p < 0.0001. ns, not significant; PAX, paroxetine; FLX, fluoxetine.

**FIGURE 2:**
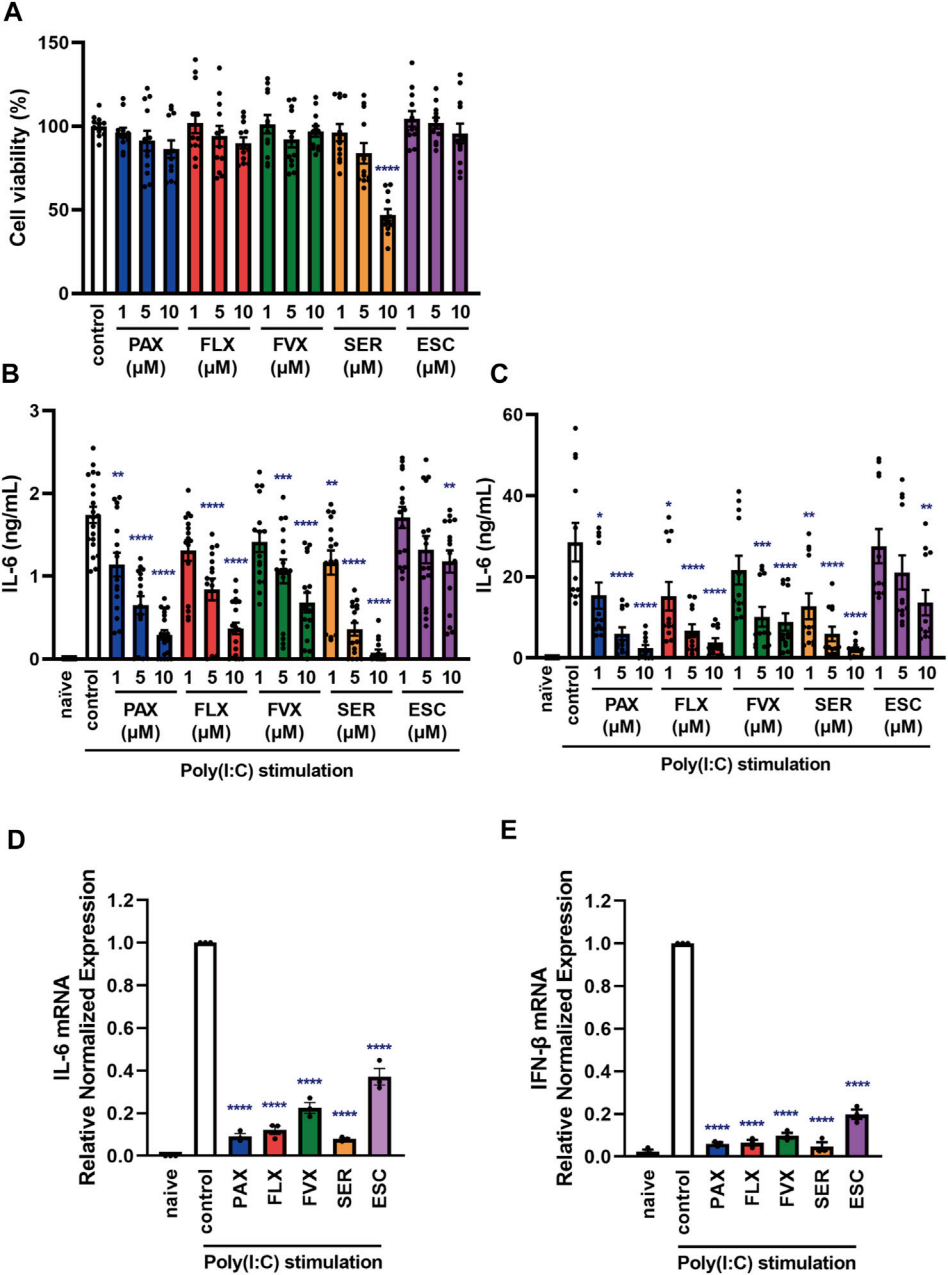
Effects of SSRIs on cytokine production induced by a TLR3 agonist in J774.1 murine macrophage cells. **(A)** J774.1 cells were incubated with each SSRI (1–10 µM) for 24 h. Cell viability was evaluated by MTT assay. **(B,C)** Cells were pre-incubated with each SSRI (1–10 µM) for 30 min and then incubated with poly(I:C) (5 μg/ml) for 6 h (B) or 24 h **(C)**. IL-6 was measured by means of ELISA. **(D,E)** Cells were pre-incubated with each SSRI (10 µM) for 30 min and then incubated with poly(I:C) (5 μg/ml) for 3 h. Expression of IL-6 or IFN-β mRNA was analyzed by real-time RT-PCR. Error bars indicate ±SE [**(A)**: n = 12, **(B)**: n = 16–20, **(C)**: n = 12, **(D,E)**: *n* = 3, three or more independent experiments]. Statistical analysis was performed by one-way ANOVA followed by Dunnett’s test. Significant differences between control and test groups are indicated by *p < 0.05, **p < 0.01, ***p < 0.001, ****p < 0.0001. PAX, paroxetine; FLX, fluoxetine; FVX, fluvoxamine; SER, sertraline; ESC, escitalopram.

**FIGURE 3:**
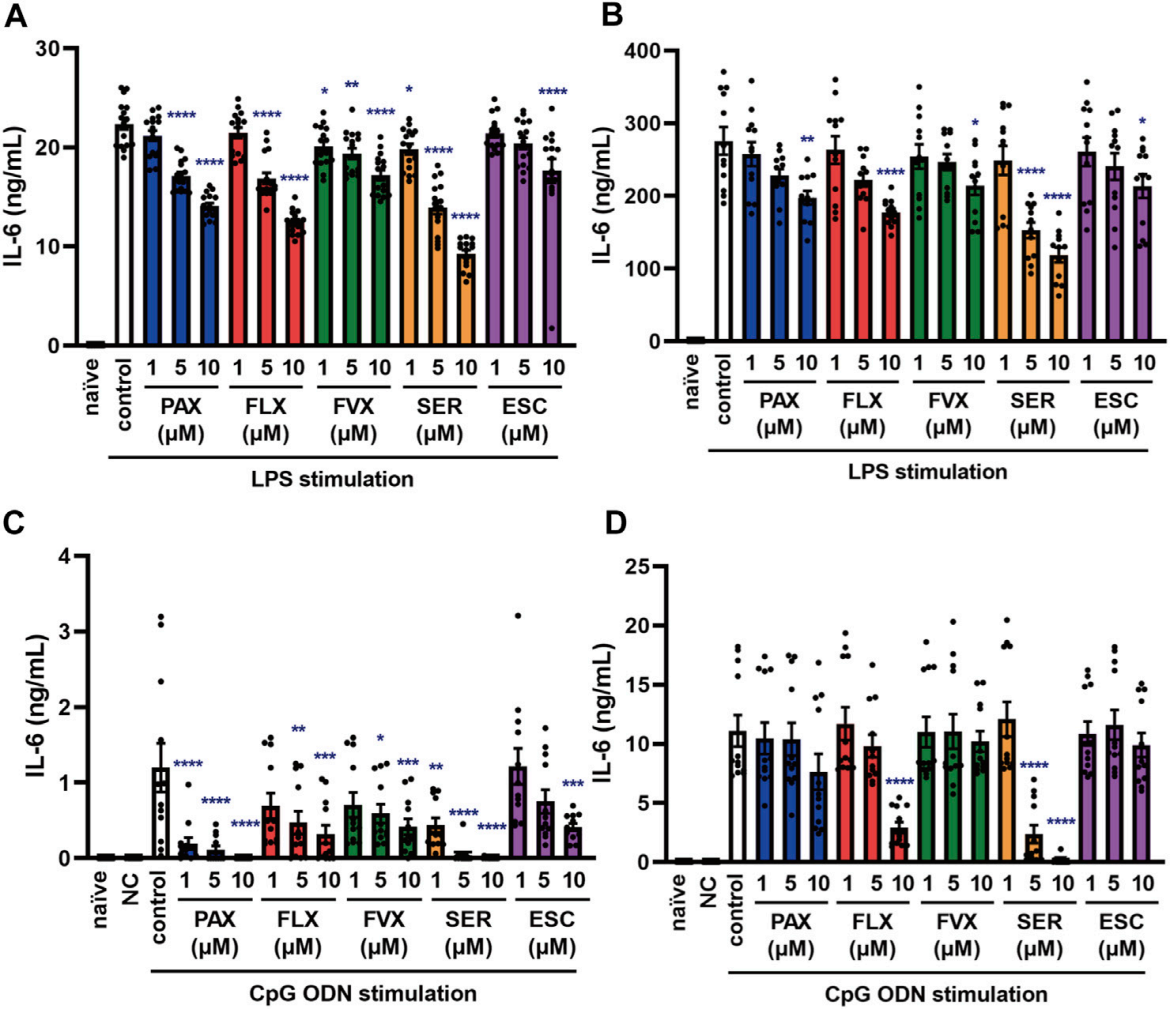
Effects of SSRIs on IL-6 production induced by TLR4 or TLR9 agonist in J774.1 murine macrophage cells. J774.1 cells were pre-incubated with each SSRI (1–10 µM) for 30 min and then incubated with LPS (1 μg/ml) for 6 h **(A)** or 24 h **(B)**, or with CpG ODN or negative control ODN (200 ng/ml) for 6 h **(C)** or 24 h **(D)**. IL-6 was measured by means of ELISA. Error bars indicate ±SE [**(A)**: *n* = 16, **(B–D)**: *n* = 12, three or more independent experiments]. Statistical analysis was performed by one-way ANOVA followed by Dunnett’s test. Significant differences between control and test groups are indicated by *p < 0.05, **p < 0.01, ***p < 0.001, ****p < 0.0001. NC, negative control ODN. PAX, paroxetine; FLX, fluoxetine; FVX, fluvoxamine; SER, sertraline; ESC, escitalopram.

**FIGURE 4:**
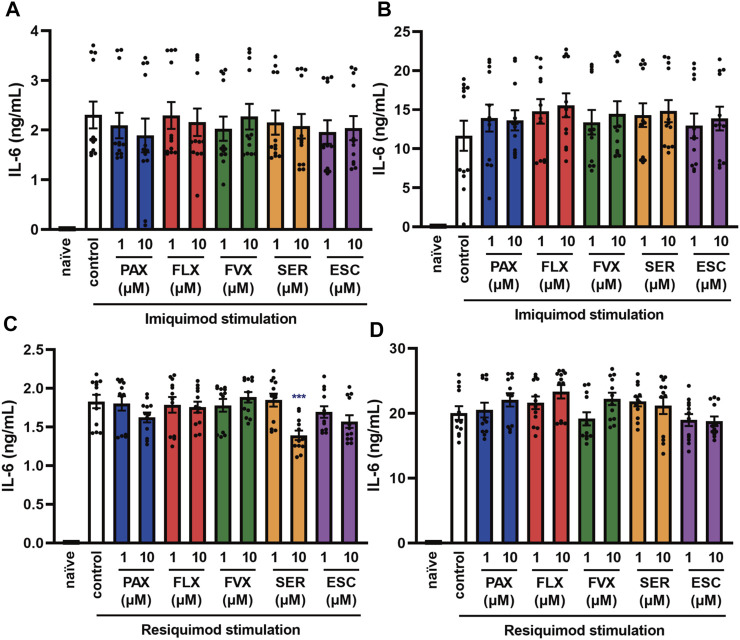
Effects of SSRIs on IL-6 production induced by TLR7 agonists in J774.1 murine macrophage cells. J774.1 cells were pre-incubated with each SSRI (1–10 µM) for 30 min and then incubated with imiquimod (1 μg/ml) for 6 h **(A)** or 24 h **(B)**, or resiquimod (10 ng/ml) for 6 h **(C)** or 24 h **(D)**. IL-6 was measured by means of ELISA. Error bars indicate ±SE (n = 12, three independent experiments). Statistical analysis was performed by one-way ANOVA followed by Dunnett’s test. Significant differences between control and test groups are indicated by ***p < 0.001. PAX, paroxetine; FLX, fluoxetine; FVX, fluvoxamine; SER, sertraline; ESC, escitalopram.

**FIGURE 5:**
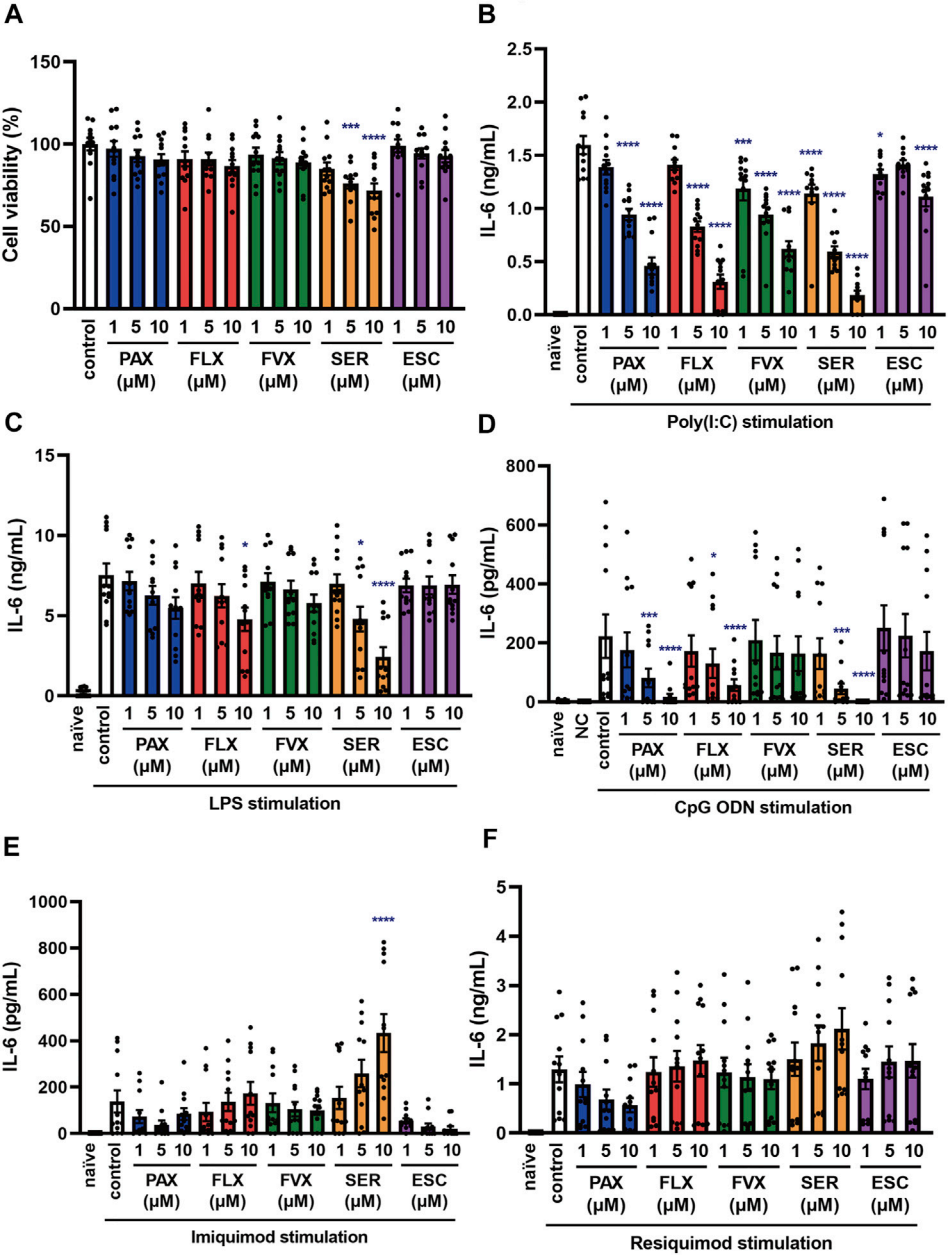
Effects of SSRIs on IL-6 production induced by TLR agonists in BMDCs. **(A)** BMDCs were incubated with each SSRI (1–10 µM) for 24 h. Cell viability was evaluated by MTT assay. **(B–F)** BMDCs were pre-incubated with each SSRI (1–10 µM) for 30 min and then incubated with poly(I:C) (10 μg/ml) **(B)**, LPS (1 μg/ml) **(C)**, CpG ODN or negative control ODN (200 ng/ml) **(D)**, imiquimod (1 μg/ml) **(E)** or resiquimod (10 ng/ml) **(F)** for 24 h. IL-6 was measured by means of ELISA. Error bars indicate ±SE [**(A–F)**: *n* = 12, three independent experiments]. Statistical analysis was performed by one-way ANOVA followed by Dunnett’s test. Significant differences between control and test groups are indicated by *p < 0.05, ***p < 0.001, ****p < 0.0001. NC, negative control ODN. PAX, paroxetine; FLX, fluoxetine; FVX, fluvoxamine; SER, sertraline; ESC, escitalopram.

**FIGURE 6:**
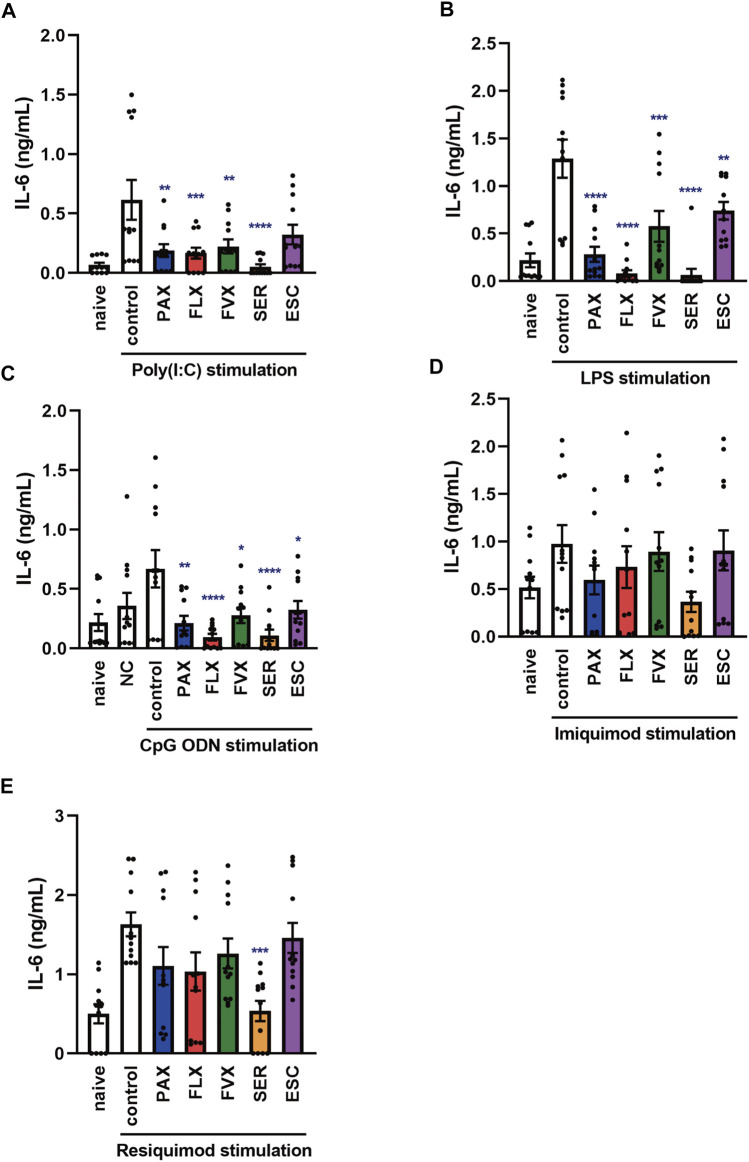
Effects of SSRIs on IL-6 production induced by TLR agonists in murine peritoneal cells. Peritoneal cells isolated from C57BL/6 mice were pre-incubated with each SSRI (1–10 µM) for 30 min and then incubated with poly(I:C) (5 μg/ml) **(A)**, LPS (1 μg/ml) **(B)**, CpG ODN or negative control ODN (200 ng/ml) **(C)**, imiquimod (1 μg/ml) **(D)** or resiquimod (10 ng/ml) **(E)** for 24 h. IL-6 was measured by means of ELISA. Error bars indicate ±SE (*n* = 12, three independent experiments). Statistical analysis was performed by one-way ANOVA followed by Dunnett’s test. Significant differences between control and test groups are indicated by *p < 0.05, **p < 0.01, ***p < 0.001, ****p < 0.0001. NC, negative control ODN. PAX, paroxetine; FLX, fluoxetine; FVX, fluvoxamine; SER, sertraline; ESC, escitalopram.

**FIGURE 7:**
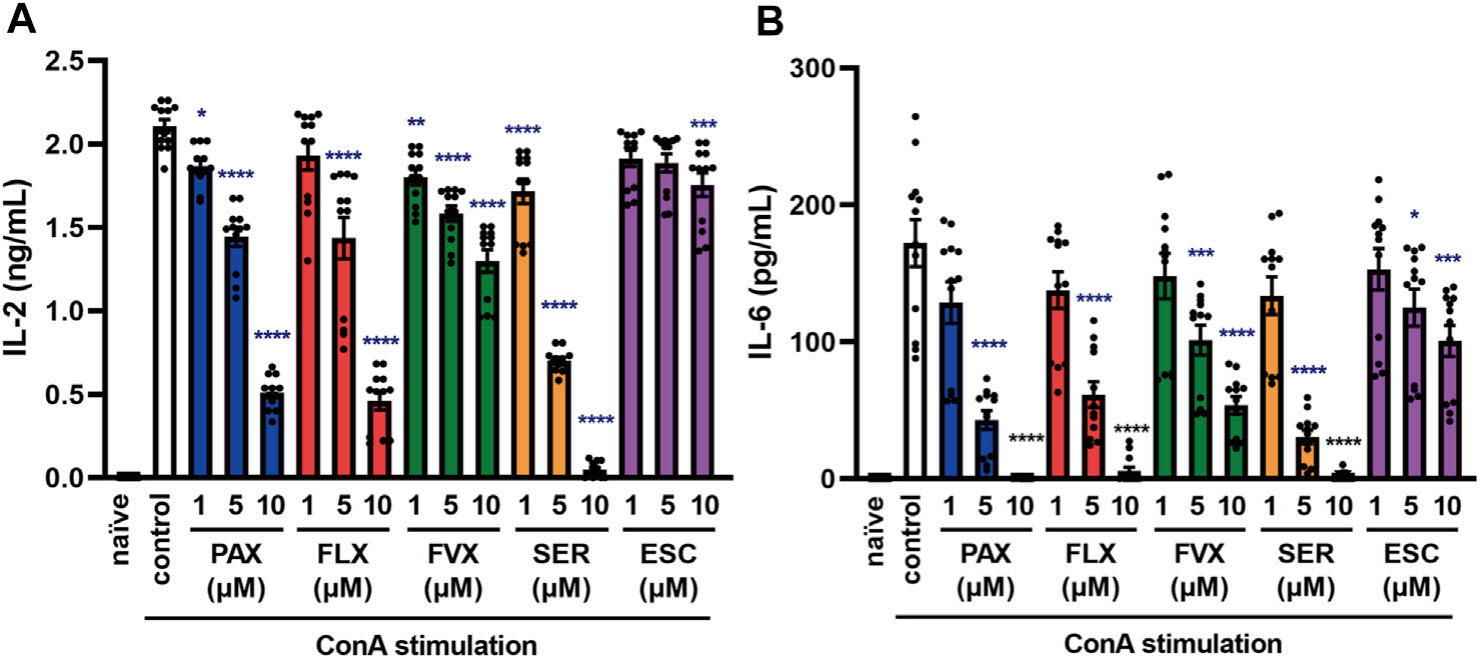
Effects of SSRIs on cytokine production induced by a T cell activator in murine splenic lymphocytes. Splenic lymphocytes of C57BL/6 mice were pre-incubated with each SSRI (1–10 µM) for 30 min and then incubated with ConA (5 μg/ml) for 24 h. IL-2 **(A)** or IL-6 **(B)** was measured by means of ELISA. Error bars indicate ±SE (*n* = 12, three independent experiments). Statistical analysis was performed by one-way ANOVA followed by Dunnett’s test. Significant differences between control and test groups are indicated by *p < 0.05, **p < 0.01, ***p < 0.001, ****p < 0.0001. ConA, Concanavalin A; PAX, paroxetine; FLX, fluoxetine; FVX, fluvoxamine; SER, sertraline; ESC, escitalopram.

**FIGURE 8:**
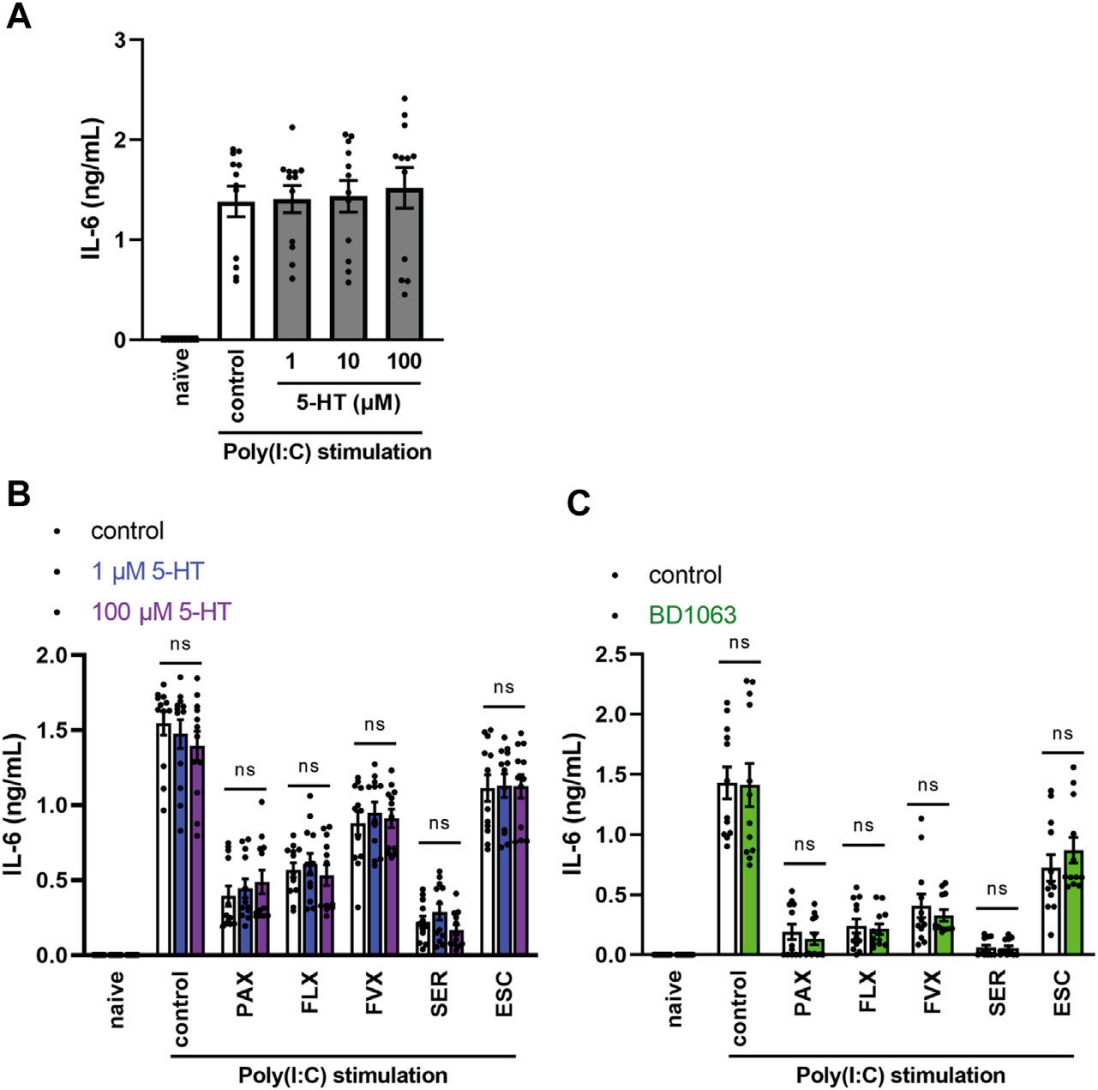
Effects of serotonin or sigma-1 receptor antagonist on the inhibition of TLR3 agonist-induced IL-6 production by SSRIs in J774.1 murine macrophage cells. **(A)** J774.1 cells were pre-incubated with serotonin (1, 10, 100 µM) for 30 min and then incubated with poly(I:C) (5 μg/ml) for 6 h **(B,C)** Cells were pre-incubated with serotonin (1, 100 µM) **(B)** or sigma-1 receptor antagonist, BD1063 (10 µM) **(C)** for 30 min, and incubated with each SSRI (10 µM) for 30 min, then incubated with poly(I:C) (5 μg/ml) for 6 h. IL-6 was measured by means of ELISA. Error bars indicate ±SE (*n* = 12, three independent experiments). Statistical analysis was performed by one-way ANOVA followed by Dunnett’s test **(A)**, two-way ANOVA followed by Dunnett’s test **(B)**, or two-way ANOVA followed by Bonferroni’s test **(C)**. 5-HT: 5-hydroxytryptamine (serotonin). PAX, paroxetine; FLX, fluoxetine; FVX, fluvoxamine; SER, sertraline; ESC, escitalopram.

**FIGURE 9:**
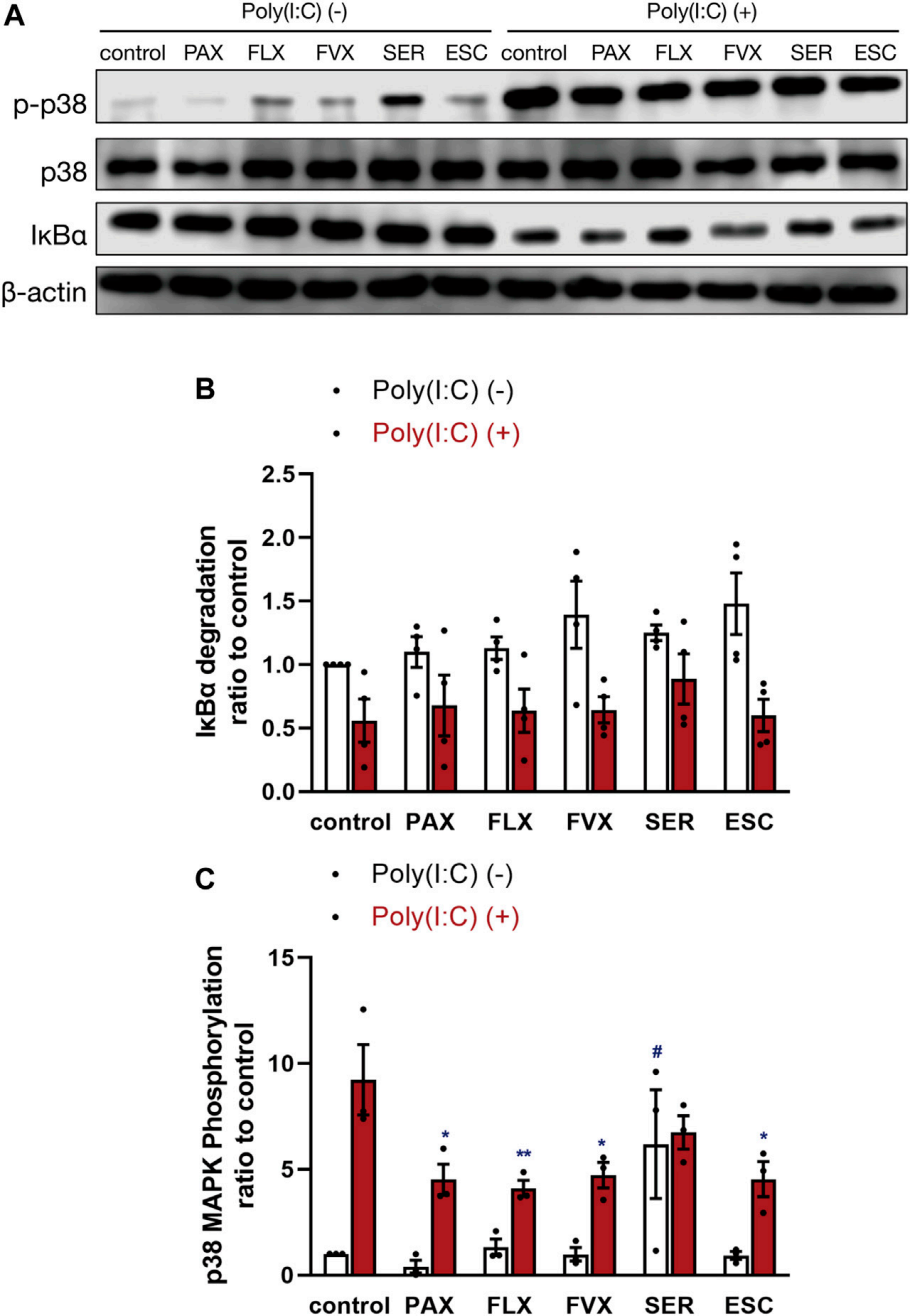
Effects of SSRIs on IκBα degradation and p38 MAPK phosphorylation induced by a TLR3 agonist in J774.1 murine macrophage cells. J774.1 cells were pre-incubated with each SSRI (10 µM) for 30 min, and then incubated with poly(I:C) (5 μg/ml) for 45 min. The β-actin blot **(A,B)** or p38 MAPK blot **(A,C)** is included as a loading control. IκBα degradation or p38 MAPK phosphorylation was analyzed by Western blotting. Error bars indicate ±SE [**(B)**: *n* = 4, four independent experiments, **(C)**: *n* = 3, three independent experiments). Statistical analysis was performed by two-way ANOVA followed by Dunnett’s test. Significant differences between poly(I:C) (−) control and test groups are indicated by ^#^ *p* < 0.01. Significant differences between poly(I:C) (+) control and test groups are indicated by *p < 0.05, **p < 0.01. PAX, paroxetine; FLX, fluoxetine; FVX, fluvoxamine; SER, sertraline; ESC, escitalopram.

**FIGURE 10:**
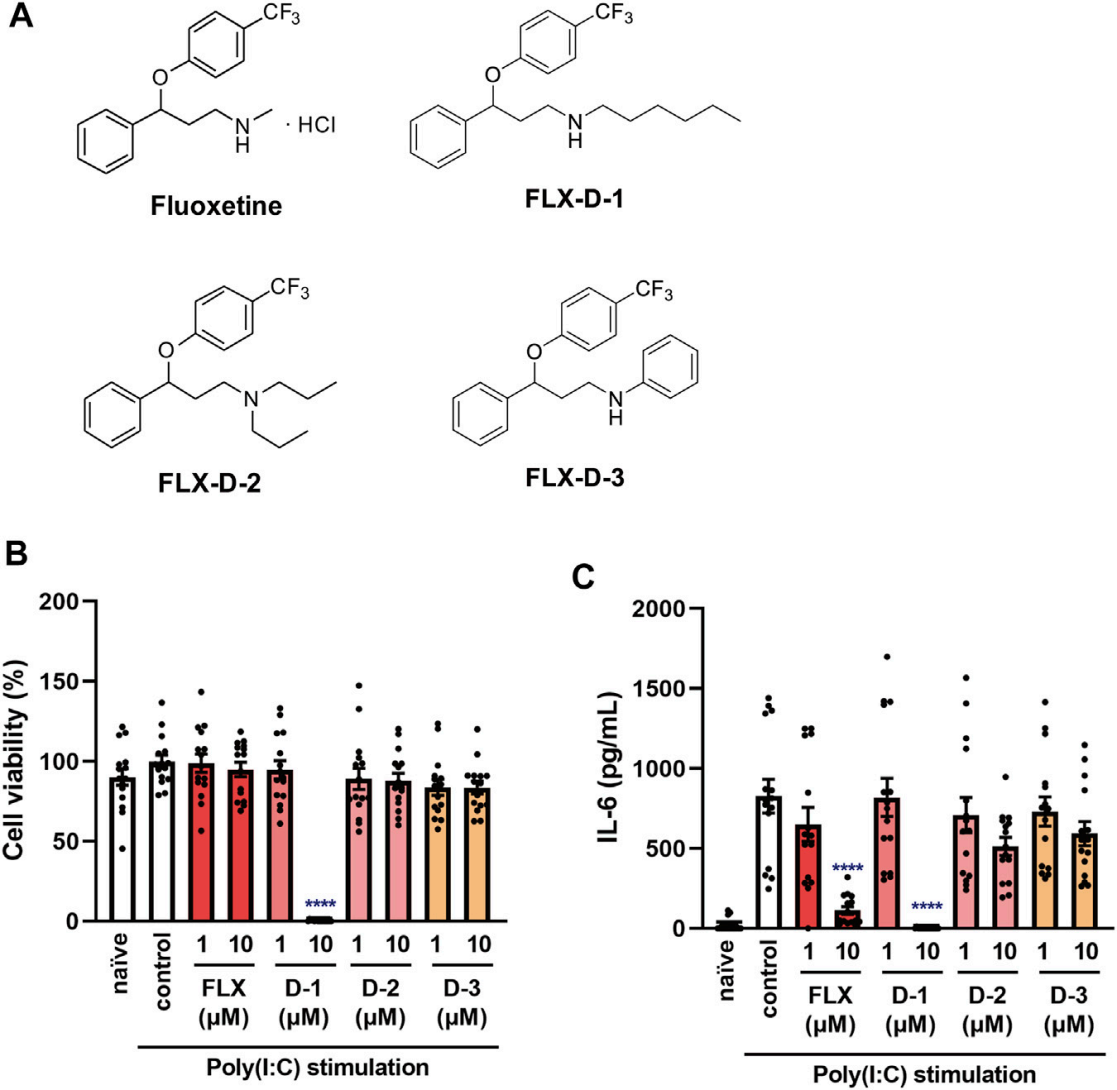
Effects of fluoxetine derivatives on cell viability and IL-6 production induced by a TLR3 agonist in J774.1 murine macrophage cells. **(A)** Structural formulas of fluoxetine and its derivatives. **(B,C)** J774.1 cells were pre-incubated with each fluoxetine derivative (FLX-D1, -D2, -D3) (1, 10 µM) for 30 min and then incubated with poly(I:C) (5 μg/ml) for 6 h. **(B)** Cell viability was evaluated by MTT assay. **(C)** IL-6 was measured by means of ELISA. Error bars indicate ±SE [**(B,C)**: *n* = 15, four independent experiments). Statistical analysis was performed by one-way ANOVA followed by Dunnett’s test. Significant differences between control and test groups are indicated by ****p < 0.0001. FLX, fluoxetine. D-1: FLX-D1, D-2: FLX-D2, D-3: FLX-D3.

Values are given as the mean ± SE. Multiple groups were compared using one-way ANOVA followed by Dunnett’s test (control group vs. other group such as drug-treated group) (Figures 1A–8A, 10) or two-way ANOVA followed by Dunnett’s test (Figures 8B, 9), or Bonferroni’s test (control group vs. other group such as drug-treated group) (Figure 8C), as implemented in GraphPad Prism version 9.0 statistical package (GraphPad Software, San Diego, CA, United States). The criterion of significance was set at *p* < 0.05.

## Results

### Selective Serotonin Reuptake Inhibitors Potently Suppress Inflammatory Cytokine Production Induced by TLR3 Agonist in J774.1 Murine Macrophage Cells

Since IL-6 is considered to aggravate various inflammatory diseases (Chousterman et al., 2017; Shimizu, 2020), controlling excessive IL-6 production is therapeutically important. TLR3 is a molecule of the innate immune system that recognizes viral-derived double-stranded RNA (dsRNA) and functions in defense against viruses (Chen et al., 2017). TLR3 is overactivated in lethal inflammatory conditions, so controlling TLR3-mediated IL-6 production is extremely important in the treatment of cytokine storms. In addition, excessive production of inflammatory cytokines due to overactivation of macrophages has been reported to contribute significantly to the aggravation of cytokine storms (Crayne et al., 2019), so we first searched for compounds suppressing IL-6 release induced by the TLR3 agonist poly(I:C) in J774.1 murine macrophage cells. In preliminary experiments, we found that paroxetine, an SSRI, markedly suppressed poly(I:C)-induced IL-6 release by J774.1 cells. Poly(I:C)-induced IL-6 secretion and increase of IL-6 mRNA expression were both significantly suppressed by pretreatment with paroxetine in a concentration-dependent manner (Figures 1A,B). In addition, we found that poly(I:C)-induced IFN-β mRNA expression was suppressed by pretreatment with paroxetine (Figure 1C), suggesting that paroxetine suppresses TLR3-induced transcription of cytokine genes. Since paroxetine inhibits not only serotonin reuptake, but also P2X4 receptor (Nagata et al., 2009), we next investigated the effects of pretreatment with the selective P2X4 receptor antagonists 5-BDBD and PSB12062, as well as another SSRI, fluoxetine. Pretreatment with these P2X4 receptor antagonists did not inhibit poly(I:C)-induced IL-6 release, but fluoxetine was significantly inhibitory, like paroxetine (Figure 1D). These results suggested that SSRIs in general might have a suppressive effect on TLR3 agonist-induced cytokine production.

To test this idea, we next examined the effects of other SSRIs, fluvoxamine, sertraline and escitalopram. Among the 5 SSRIs (1–10 µM), only 10 µM sertraline had a significant effect on the viability of J774.1 cells at 24 h (Figure 2A). Though 10 µM sertraline significantly decreased cell viability at 24 h, 10 µM sertraline did not significantly decrease cell viability within 6 h in J774.1 cells (data not shown). We found that the secretion of IL-6 was suppressed by pretreatment with all of these SSRIs at 6 h (Figure 2B) and 24 h (Figure 2C) after poly(I:C) treatment, though escitalopram had a weaker effect than the others. Similarly, all of the SSRIs suppressed poly(I:C)-induced IL-6 (Figure 2D) and IFN-β (Figure 2E) mRNA expression. These results suggest that SSRIs inhibit the TLR3-mediated production of cytokines such as IL-6.

### Selective Serotonin Reuptake Inhibitors Also Suppress IL-6 Production Induced by TLR4 and TLR9 Agonists, but Not by TLR7 Agonists in J774.1 Murine Macrophage Cells

Sepsis is a systemic reaction syndrome arising after infection, and the most frequent causative agent is bacteria (Chousterman et al., 2017; Kuzmich et al., 2017; Rudd et al., 2020). There has been a report comparing the effect of 5 SSRIs on LPS-induced production of TNF-α, but no report comparing their effects on IL-6 production (Tynan et al., 2012). Thus, we next examined the effect of these SSRIs on IL-6 release induced by LPS, which is a TLR4 agonist. IL-6 release was partially suppressed by all of the SSRIs (Figures 3A,B), but these SSRIs were less inhibitory towards TLR4-mediated IL-6 production than towards TLR3-mediated IL-6 production.

TLR9 recognizes unmethylated cytosine-phosphate-guanosine (CpG) type DNA existing in bacterial and viral genomes (Briard et al., 2020). Though it has been reported that fluoxetine and citalopram suppress TLR9 agonist-induced cytokine production (Sacre et al., 2010), the effects of other SSRIs have not been reported. Therefore, we next examined the effect of the 5 SSRIs on IL-6 release induced by CpG ODN, a TLR9 agonist. IL-6 secretion at 6 h after CpG ODN treatment was suppressed by all of the SSRIs (Figure 3C), while that at 24 h was suppressed only by high-dose fluoxetine or sertraline (Figure 3D). These results suggest that SSRIs suppress TLR9-mediated IL-6 production only in the early phase, so they might be candidate therapeutic agents for the acute phase of DNA virus infections.

TLR7 and TLR8 recognize viral single-stranded RNA (ssRNA) (Chen et al., 2017). To elucidate the effects of SSRIs on TLR7-mediated cytokine production, we next investigated IL-6 secretion induced by the TLR7 agonist imiquimod and the TLR7/8 agonist resiquimod. TLR8 is expressed in mice, but does not respond to ssRNA or other TLR8 ligands, so it appears not to function in mice (Heil et al., 2004). We found that the SSRIs had little effect on IL-6 release induced by imiquimod (Figures 4A,B) or resiquimod (Figures 4C,D). Only high-dose sertraline partially attenuated IL-6 release induced by resiquimod. These results suggest that SSRIs in general have little or no effect on TLR7-mediated production of IL-6 in macrophages.

### Effects of Selective Serotonin Reuptake Inhibitors on Toll-Like Receptor Agonist-Induced IL-6 Production in Murine Bone Marrow-Derived Dendritic Cells

Dendritic cells are known to be involved in the exacerbation of cytokine storms, as well as macrophages, so controlling their activation could be one of the targets of cytokine storm therapy (Crayne et al., 2019). Thus, we examined the effects of SSRIs on IL-6 production induced by various TLR agonists in murine BMDCs. Among the 5 SSRIs (1–10 µM) only high doses (5–10 µM) of sertraline had a significant effect on the viability of BMDCs (Figure 5A), which is similar to the results obtained with J774.1 cells. Pretreatment with all of the SSRIs suppressed the TLR3 agonist-induced secretion of IL-6 by BMDCs (Figure 5B) but the inhibitory effect of escitalopram was weak, which is also similar to the results obtained in J774.1 cells. Moreover, pretreatment with most of the SSRIs suppressed TLR4- or TLR9-mediated IL-6 release from BMDCs. The IL-6 secretion from BMDCs induced by stimulation with a TLR4 agonist was significantly suppressed by fluoxetine and sertraline (Figure 5C), while the secretion induced by stimulation with a TLR9 agonist was significantly suppressed by paroxetine, fluoxetine and sertraline (Figure 5D). On the other hand, the effects of the SSRIs on TLR7-mediated IL-6 release were all different, and also differed considerably from the effects observed in J774.1 cells described above. Sertraline significantly amplified the IL-6 release induced by imiquimod (Figure 5E), and also tended to amplify that induced by resiquimod (Figure 5F). These results indicate that the effects of SSRIs on TLR3-, TLR4- and TLR9-mediated IL-6 production are similar in macrophages and dendritic cells, but the effects of SSRIs on TLR7-mediated IL-6 production differ markedly between J774.1 cells and BMDCs, and furthermore, the effects in BMDCs may vary from SSRI to SSRI.

### Effects of Selective Serotonin Reuptake Inhibitors on Toll-Like Receptor Agonist-Induced IL-6 Production in Peritoneal Cells Isolated From Mice

Next, to investigate the effects of SSRIs on IL-6 production in naïve cells in the living body, we focused on TLR-mediated IL-6 production in murine peritoneal cells of C57BL/6 mice, which include peritoneal macrophages. Pretreatment with the SSRIs except for escitalopram significantly suppressed the TLR3 agonist-induced secretion of IL-6 by murine peritoneal cells (Figure 6A). Also, pretreatment with all of the SSRIs suppressed IL-6 production induced by TLR4 or TLR9 agonist (Figures 6B,C). On the other hand, TLR7-mediated IL-6 release was significantly attenuated only by sertraline among the SSRIs (Figures 6D,E), which is similar to the results in J774.1 cells, but different from the results in BMDCs. These findings suggest that SSRIs are able to inhibit IL-6 production stimulated by TLR3, TLR4 or TLR9 agonists, regardless of cell type. On the other hand, the effect of SSRIs on TLR7-mediated IL-6 production appears to differ depending on the cell type and the SSRI employed.

### Selective Serotonin Reuptake Inhibitors Suppress Concanavalin A-Induced Cytokine Production From Murine Splenic Lymphocytes

When foreign microorganisms, such as viruses or bacteria invade the body, immune response signals lead to the activation of T cell receptor (TCR) (Dinkel et al., 2018). The abnormal activation of T cells is also associated with the aggravation of cytokine storms (Chousterman et al., 2017; Shimizu, 2020), so controlling T cell activation is important in the treatment of inflammatory diseases. To understand the effect of SSRIs on TLR-independent cytokine release in lymphocytes, including T cells, we examined the effects of SSRIs on concanavalin A (ConA)-induced cytokine production in splenic lymphocytes isolated from C57BL/6 mice. Interestingly, pretreatment with all of the SSRIs significantly suppressed not only IL-6 release but also IL-2 release induced by ConA from murine splenic lymphocytes (Figure 7). These results suggest that SSRIs inhibit the production of IL-6 and IL-2 caused by ConA-induced T cell activation, which occurs independently of TLR activation.

### Selective Serotonin Reuptake Inhibitors Inhibit TLR3-Mediated IL-6 Release Independently of Extracellular Serotonin and Sigma-1 Receptors

The antidepressant effects of SSRIs are exerted via inhibition of the serotonin transporter (SERT), thereby increasing extracellular serotonin levels (Hiemke and Härtter, 2000; Lochmann and Richardson, 2019). We investigated whether increased extracellular serotonin is involved in the inhibitory effect of SSRIs on TLR3-mediated IL-6 release. However, pretreatment with serotonin had no effect on IL-6 release from J774.1 cells treated with TLR3 agonist (Figure 8A). Similarly, serotonin had no effect on the inhibition of TLR3-mediated IL-6 production by SSRIs (Figure 8B). These results suggest that SSRIs inhibit TLR3 signaling through a serotonin-independent mechanism.

Sigma-1 receptor (S1R) is a single transmembrane receptor expressed mainly on the endoplasmic reticulum (ER) membrane (Merlos et al., 2017; Rosen et al., 2019; Lenze et al., 2020; Hashimoto, 2021). S1R is involved in the regulation of cytokine production through its interaction with inositol-requiring enzyme 1α (IRE1α), an ER stress sensor protein (Rosen et al., 2019; Hashimoto, 2021). S1R and IRE1α are thought to be deeply involved in the pathogenesis of various peripheral and central nervous system diseases, including chronic pain, psychiatric disorders, and cognitive dysfunction (Merlos et al., 2017). SSRIs have a high affinity for S1Rs (Hashimoto, 2021), and it was proposed that S1Rs may be involved in the suppression of COVID-19 severity by fluvoxamine (Lenze et al., 2020; Hashimoto, 2021; Reis et al., 2022). Therefore, we next examined whether the S1R-agonistic effect of SSRIs is involved in the inhibitory effects of SSRIs on TLR3-mediated IL-6 production. However, S1R antagonist did not alter the inhibitory effect of SSRIs on TLR3-mediated IL-6 release (Figure 8C). These results suggest that SSRIs inhibit TLR3 signaling independently of S1R-agonistic action.

### Effects of Selective Serotonin Reuptake Inhibitors on TLR3 Agonist-Induced Intracellular Signaling

Since it is well known that TLR3-stimulated production of inflammatory cytokines is mediated by activation of NF-κB and MAPKs (Zhu et al., 2010; Chen et al., 2017), we investigated the effects of SSRIs on degradation of IκBα, a regulator of NF-κB activation, and on p38 MAPK phosphorylation. IκBα degradation induced by poly(I:C) was unaffected by pretreatment with the 5 SSRIs (Figures 9A,B). On the other hand, p38 MAPK phosphorylation induced by poly(I:C) was inhibited by paroxetine, fluoxetine, fluvoxamine, and escitalopram, while sertraline enhanced it without poly(I:C) treatment (Figures 9A,C). These results suggest that SSRIs may suppress TLR3-mediated IL-6 production independently of NF-κB and p38 MAPK.

### Effects of Fluoxetine Derivatives on TLR3 Agonist-Induced IL-6 Production and Viability of J774.1 Murine Macrophage Cells

Since we found that fluoxetine potently inhibits IL-6 production induced by various stimuli and in various cells, as well as having low cellular toxicity, we considered that fluoxetine might be the most suitable anti-inflammatory candidate to suppress cytokine storms among the 5 SSRIs. Therefore, we examined the structure-activity relationship of fluoxetine and attempted to optimize its structure by synthesizing several derivatives (FLX-D-1, 2, and 3) (Figure 10A). A derivative of fluoxetine with a longer carbon chain attached to the amine (FLX-D-1) was found to significantly reduce the viability of J774.1 cells (Figure 10B). Although FLX-D-1 inhibited the IL-6 release induced by poly(I:C) (Figure 10C), this effect may be attributable to its cytotoxicity. FLX-D-2 and FLX-D-3 did not exhibit cytotoxicity, and did not affect the IL-6 release induced by poly(I:C) (Figures 10B,C). These results indicate that the substituent at the nitrogen atom in fluoxetine greatly influences both cytotoxic activity and IL-6 production. The small methyl group of fluoxetine appears to be critical for the inhibition of the IL-6 release and for low cytotoxicity, whereas a large or bulky hydrophobic substituent is unfavorable for the inhibition of IL-6 production.

## Discussion

This is the first study designed to identify which SSRI would be the most promising anti-inflammatory agent to suppress cytokine storms, based on a comparison of the effects of the 5 SSRIs on IL-6 production by various immune stimuli. Our key findings are as follows. First, SSRIs in general suppress IL-6 production induced by TLR3, TLR4 and TLR9 agonists in macrophages and dendritic cells. Second, the order of the inhibitory effects of SSRIs on TLR-mediated IL-6 production is TLR3 > TLR9 > TLR4. Third, sertraline specifically amplifies TLR7 agonist-induced IL-6 production in dendritic cells, but not in macrophages. Fourth, SSRIs suppress ConA-induced cytokine production in T lymphocytes. Fifth, the order of activity of SSRIs in suppressing IL-6 production is sertraline > paroxetine, fluoxetine > fluvoxamine > escitalopram. Our findings suggest that, because of having potent inhibitory effect of IL-6 production and low cytotoxicity, fluoxetine may be the most promising candidate among the 5 SSRIs for cytokine storm treatment.

We found that all 5 SSRIs suppress IL-6 production induced by poly(I:C), a TLR3 agonist. Also, the SSRIs inhibited IL-6 production induced by poly(I:C) more potently than that induced by other TLR agonists, demonstrating the high selectivity of SSRIs for TLR3 signaling. It has already been reported that fluoxetine, sertraline, and citalopram inhibit the TLR3 pathway (Sacre et al., 2010; Zhu et al., 2010). Our study confirmed that SSRIs in general modulate TLR3 signaling. It has been reported that the suppressive effect on TLR3 expression is greater than that on other TLRs in peripheral blood mononuclear cells (PBMCs) of patients taking antidepressants, mostly SSRIs (Hung et al., 2016), which is consistent with the idea that SSRIs selectively modulate TLR3 signaling.

Our results showed that SSRIs suppressed IL-6 production induced by CpG ODN, a TLR9 agonist. Also, we show here that the inhibitory effects of SSRIs on CpG ODN-induced IL-6 production differed from SSRI to SSRI. This is different from the results of the other TLR-mediated IL-6 production. A possible reason is that the activation of TLR9 might induce a secondary signaling to induce secondary IL-6 production between 6 and 24 h after its stimulation. Only fluoxetine and sertraline might be able to suppress this signaling pathway. Therefore, SSRIs may be more suitable for use as anti-inflammatory agents to treat RNA virus-induced inflammation, rather than DNA virus-induced inflammation.

In this study, SSRIs did not affect TLR7 agonist-induced IL-6 release from J774.1 murine macrophage cells or murine peritoneal cells. These results are contrary to a previous report (Sacre et al., 2010). It is possible that the apparent discrepancy can be explained in terms of differences in cell sensitivity, or the higher concentration they used. Meanwhile, it has been reported that high doses of sertraline (10–30 µM) have no effect on cytokine production via TLR7/8 signaling (Zhu et al., 2010), which at least suggests that SSRIs are not highly selective for TLR7 signaling.

Notably, sertraline amplified the production of IL-6 induced by TLR7 agonists in murine dendritic cells, not in macrophages. Among the 5 SSRIs, only sertraline has been reported to have an antagonistic effect on S1Rs (Hashimoto, 2021) and to induce ER stress (Chen et al., 2014). It has been reported that gp96, one of the ER stress response proteins, is more likely to be induced in dendritic cells than macrophages (Wolfram et al., 2013). Therefore, it is possible that dendritic cells are more sensitive to ER stress, which might amplify the ER stress-inducing effect of sertraline. Among the 5 SSRIs, we found that sertraline had the greatest effect in reducing cell viability, but it also had the strongest inhibitory effect on IL-6 production, thus it can be considered as a double-edged sword.

Furthermore, we found that the SSRIs partially suppress LPS-induced IL-6 release from murine macrophages and dendritic cells. However, the order of the inhibitory effects of SSRIs on cytokine production is somewhat different between the previous report on LPS-induced TNF-α production in BV-2 murine microglial cells and our results (Tynan et al., 2012). It seems likely that the effects of SSRIs may vary depending on the kinds of cells and cytokines. Some SSRIs have been reported to suppress LPS-induced inflammation *in vivo* (Roumestan et al., 2007; Ohgi et al., 2013; Dong et al., 2016; Rosen et al., 2019). Therefore, SSRIs appear to have a robust effect on TLR4 signaling.

We also found here that SSRIs inhibit cytokine production induced by ConA in murine splenic lymphocytes, suggesting that SSRIs inhibit also TLRs-independent cytokine production, and that the target(s) of their inhibitory effect may be molecules commonly expressed on macrophages, dendritic cells, and T cells. Paroxetine significantly suppresses IL-2 production induced by TCR activation in T cells in human PBMCs via G protein-coupled receptor kinase-2 (GRK2) inhibition (Dinkel et al., 2018). However, it seems that SSRIs in general may not inhibit GRK2; at least fluoxetine did not have this effect (Thal et al., 2012). On the other hand, some SSRIs significantly inhibited the proliferation of TCR-stimulated T cells in human PBMCs (Gobin et al., 2013). Thus, SSRIs affect T cell activation, supporting their potential value as anti-inflammatory agents.

Peripheral serotonin has been suggested to exert immunomodulatory effects. However, we found that serotonin had no effect on TLR3-mediated IL-6 production, whereas SSRIs were inhibitory, suggesting that SSRIs’ inhibitory effect may be independent of serotonin signaling. These results are consistent with several previous reports (Sacre et al., 2010; Durairaj et al., 2015). In addition, our results are inconsistent with the intensity of the SERT’s inhibitory effects of SSRIs (Hiemke and Härtter, 2000), also supporting that the inhibitory effects of SSRIs on IL-6 production are independent for serotonin signaling.

In this context, recent reports that fluoxetine and fluvoxamine suppressed the exacerbation of COVID-19 (Lenze et al., 2020; Németh et al., 2021; Reis et al., 2022), could be explained in terms of the inhibitory effects on cytokine production. It was suggested that the stimulation of S1R is involved in the case of fluvoxamine (Lenze et al., 2020). However, we found that an S1R antagonist did not modulate the inhibitory effect of SSRIs on TLR3 agonist-induced IL-6 production. In addition, although recent reports indicate that fluvoxamine has the highest affinity for S1R among the SSRIs (Hashimoto, 2021), our results indicate that the inhibitory effect of fluvoxamine on IL-6 production is not as potent as those of other SSRIs. Overall, these results suggest that the clinically observed effect involves some mechanism(s) other than action on S1R, at least in part.

We found that some of the SSRIs regulate poly(I:C)-stimulated activation of p38 MAPK, but not that of NF-κB. These results are consistent with several recent reports (Zhu et al., 2010; Liu et al., 2011), but those effects of SSRIs seem to be only partially related to their inhibitory effects on IL-6 production. Recent reports indicate that sertraline did not affect TLR3-NF-κB signaling (Zhu et al., 2010), and SSRIs did not affect phosphorylation and nuclear translocation of NF-κB (p65) induced by cytokine mixture (He et al., 2021). On the other hand, a report has shown that fluoxetine inhibits LPS-induced NF-κB activation in BV-2 murine microglial cells (Liu et al., 2011). However, there was a report that the increase of lacrimal gland serotonin levels in depressed patients treated with SSRIs promotes production of inflammatory cytokines through activation of NF-κB (Zhang et al., 2019). Thus, the effect of SSRIs on NF-κB activation may vary depending on the stimulus and cell type. On the other hand, recent reports indicate that fluoxetine inhibits LPS-induced phosphorylation of p38 MAPK (Liu et al., 2011), whereas paroxetine has no effect (Liu et al., 2014). What is surprising is that only sertraline induced phosphorylation of p38 MAPK without poly(I:C) treatment. This effect of sertraline might contribute to its high cytotoxicity.

It is widely known that TLR3 signals through TIR-domain-containing adapter-inducing interferon-β (TRIF), TLR4 through TRIF and myeloid differentiation factor 88 (MyD88), and TLR7 and TLR9 through MyD88 (Chen et al., 2017; Kuzmich et al., 2017; Briard et al., 2020). Since SSRIs suppressed both TRIF-dependent and MyD88-dependent TLR signaling, SSRIs might not directly affect the common downstream signals of the TLRs. It might be possible that SSRIs may affect other molecules such as unc-93 homolog B1 (UNC93B1) or acid sphingomyelinase (ASM) as described below.

A possible explanation that SSRIs do not have the same effects on inflammation induced via different TLRs would be the involvement of UNC93B1. UNC93B1 is a polytopic membrane protein that allows nucleic acid sensing TLRs to move from the ER to the endosomal compartment, facilitating their signaling (Majer et al., 2019). Dissociation of TLR3 or TLR9 from UNC93B1 in the endosome is essential for their signaling, while this is not the case for TLR7 (Majer et al., 2019). This seems consistent with the idea that SSRIs may block the dissociation of UNC93B1 and TLRs in endosomes.

Among the potential targets of SSRIs, another candidate is ASM. ASM is a sphingomyelinase with an acidic optimal pH that degrades sphingomyelin, a phospholipid, into ceramide and phosphocholine (Kornhuber et al., 2011; Perry et al., 2014; Gulbins et al., 2015). Excessive activity of ASM is involved in depression, and various antidepressants including SSRIs inhibit the activity of ASM (Gulbins et al., 2015). Furthermore, ASM is involved in the induction of proinflammatory cytokines (Perry et al., 2014). Since the reported order of ASM-inhibitory activity by SSRIs (sertraline > fluoxetine > paroxetine > fluvoxamine > citalopram) (Kornhuber et al., 2011) is similar to the order of the inhibition of TLR-induced IL-6 production found in this study, inhibition of ASM may be involved in the anti-inflammatory effect of SSRIs.

Among the SSRIs examined in this study, fluoxetine was a potent inhibitor of cytokine production induced by various stimuli and in various cells. Sertraline had a greater ability to inhibit IL-6 production than any other SSRI, but it reduced cell viability and actually amplified TLR7-mediated IL-6 production in dendritic cells. Paroxetine exerts anti-inflammatory effects via inhibition of GRK2 and P2X4 receptors (Nagata et al., 2009; Murga et al., 2019), and we found that it showed similar inhibitory effects on IL-6 production to fluoxetine. However, high doses of paroxetine enhance production of the inflammatory cytokine IL-1β (Kabiri et al., 2020). On the other hand, fluvoxamine and escitalopram did not potently inhibit cytokine production, compared to fluoxetine. Overall, we suggest that fluoxetine may be the preferred SSRI for further evaluation as an agent to ameliorate cytokine storms.

In order to explore the structural requirements for inhibition of IL-6 production, we also synthesized three fluoxetine derivatives (FLX-D-1, 2 and 3). FLX-D-1 and FLX-D-3 have hexyl and phenyl groups instead of the methyl group in fluoxetine, respectively. FLX-2 has two *n*-propyl groups at the nitrogen atom. To our surprise, only fluoxetine inhibited IL-6 production without exhibiting serious cytotoxicity. These findings suggest that the methyl group at the nitrogen atom in fluoxetine plays a critical role in the inhibition of IL-6 production, and may be helpful in structural optimization of fluoxetine for anti-inflammatory activity.

To demonstrate the usefulness of SSRIs as anti-inflammatory agents, it is necessary to investigate the other inflammatory responses, such as immune cell mobilization *in vitro* and *in vivo*. Previous report suggests that SSRIs such as fluoxetine and fluvoxamine did not affect cell migration of human polymorphonuclear cells (Sacerdote et al., 1994). However, other report suggests that fluoxetine induces slow cell rolling of leukocytes on endothelium in mice (Herr et al., 2014). On the other hand, extravascular migration of leukocytes in mice with aseptic peritonitis was not affected by acute fluoxetine treatment. (Herr et al., 2014). Considering these results, SSRIs might affect immune cell mobilization *in vitro* or *in vivo*. Thus, further study will be needed to investigate the effect of SSRI on other immune cell responses including cell mobilization.

In conclusion, our results indicate that SSRIs significantly suppress IL-6 production induced by various TLR agonists in murine macrophages and dendritic cells, as well as TLR-independent cytokine production in murine lymphocytes. Since SSRIs potently inhibit IL-6 production via TLR3, SSRIs may be effective as therapeutic agents against cytokine storms induced by viral infection. There are currently no anti-inflammatory drugs used specifically for inflammation triggered by viral infection, so the diversion of SSRIs for this purpose would be valuable. However, the inhibitory effects differ among the SSRIs. Overall, our findings suggest that fluoxetine might be the preferred SSRI for further evaluation as an anti-inflammatory drug to treat cytokine storms.

## Data Availability

The original contributions presented in the study are included in the article/supplementary materials, further inquiries can be directed to the corresponding author.
